# Sca-1**+** Cells from Fetal Heart with High Aldehyde Dehydrogenase Activity Exhibit Enhanced Gene Expression for Self-Renewal, Proliferation, and Survival

**DOI:** 10.1155/2015/730683

**Published:** 2015-03-10

**Authors:** Devaveena Dey, Guodong Pan, Nadimpalli Ravi S. Varma, Suresh Selvaraj Palaniyandi

**Affiliations:** ^1^Department of Radiology, Stanford University School of Medicine, Stanford, CA 94305, USA; ^2^Institute for Stem Cell Biology and Regenerative Medicine, Stanford University School of Medicine, Stanford, CA 94305, USA; ^3^Division of Hypertension and Vascular Research, Department of Internal Medicine, Henry Ford Health System, Detroit, MI 48202, USA; ^4^Cellular and Molecular Imaging Laboratory, Department of Radiology, Henry Ford Health System, Detroit, MI 48202, USA; ^5^Department of Chemical and Systems Biology, Stanford University, Stanford, CA 94305, USA; ^6^Department of Physiology, Wayne State University, Detroit, MI 48202, USA

## Abstract

Stem/progenitor cells from multiple tissues have been isolated based on enhanced activity of cytosolic aldehyde dehydrogenase (ALDH) enzyme. ALDH activity has emerged as a reliable marker for stem/progenitor cells, such that ALDH^bright/high^ cells from multiple tissues have been shown to possess enhanced stemness properties (self-renewal and multipotency). So far though, not much is known about ALDH activity in specific fetal organs. In this study, we sought to analyze the presence and activity of the ALDH enzyme in the stem cell antigen-1-positive (Sca-1+) cells of fetal human heart. Biochemical assays showed that a subpopulation of Sca-1+ cells (15%) possess significantly high ALDH1 activity. This subpopulation showed increased expression of self-renewal markers compared to the ALDH^low^ fraction. The ALDH^high^ fraction also exhibited significant increase in proliferation and pro-survival gene expression. In addition, only the ALDH^high^ and not the ALDH^low^ fraction could give rise to all the cell types of the original population, demonstrating multipotency. ALDH^high^ cells showed increased resistance against aldehyde challenge compared to ALDH^low^ cells. These results indicate that ALDH^high^ subpopulation of the cultured human fetal cells has enhanced self-renewal, multipotency, high proliferation, and survival, indicating that this might represent a primitive stem cell population within the fetal human heart.

## 1. Introduction

Stem cell antigen-1-positive (Sca-1+) cells from adult mouse hearts were shown to demonstrate increased proliferation and stemness along with potential to differentiate into multiple cardiac cell lineages [1–3]. Smits et al. have successfully isolated Sca-1+ cells from adult human heart and further demonstrated their ability to differentiate into cardiomyocytes [4]. These studies unequivocally suggest that Sca-1+ cells isolated from cardiac tissue are a subset of cardiac progenitor cells. Over the years, several approaches and strategies have been developed to enhance regeneration capacity of stem/progenitor cells by improving means of identification, expansion, pluripotency, self-renewal, and survival of these cells [5]. For instance, circulating progenitor cells, umbilical cord blood cells (UCBCs), hematopoietic stem cells (HSCs), and tissue-specific stem/progenitor cells are being identified based on aldehyde dehydrogenase (ALDH) activity [6–12]. Instead of solely relying on presence of cell surface markers, which may sometimes vary upon experimental processing during cell isolation, the functional cytosolic ALDH (ALDH1) activity assay is becoming more reliable and widely used [7, 13]. ALDH^high^ cells from multiple tissues have been shown to possess enhanced stemness properties, specifically self-renewal and differentiation [7, 11, 13]. ALDH^high^ stem cells are a small population of cells (0.5–5%) highly enriched for pluripotency [14–16]. In fact ALDH^high^ stem cells isolated from the blood are in clinical trials for ischemic heart failure [17]. Therefore in this study, we hypothesized that among the Sca-1+ cells from the human fetal heart, ALDH^high^ cells exhibit high self-renewal capacity, stemness, survival, and proliferation capacity compared to ALDH^low^ cells.

## 2. Materials and Methods

### 2.1. Isolation and Expansion of Fetal Sca-1+ Cells

To isolate fetal human Sca-1+ cells, anti-mouse Sca-1 antibody based magnetic separation was used, as described in a previous protocol [4]. The study protocol used here was approved by the Stanford Institutional Review Board. In brief, human fetal hearts (StemExpress, Diamond Springs, CA) were perfused using a Lagendorff apparatus, using Tyrode solution containing collagenase. Following this, fetal Sca-1+ cells were isolated by magnetic cell sorting (MACS, Miltenyi Biotec, Sunnyvale, CA), using Sca-1-coupled magnetic beads, according to the manufacturer's protocol. Sca-1+ cells were eluted from the column by washing with PBS supplemented with 0.5% bovine serum albumin and 2 mM EDTA. The eluted Sca-1+ cells were cultured on 0.1% gelatin-coated dishes in M199 (Gibco)/EGM-2 (3 : 1) media, supplemented with 10% FBS (Gibco), 10 ng/mL basic fibroblast growth factor (bFGF), 5 ng/mL epithelial growth factor (EGF), 5 ng/mL insulin-like growth factor (IGF-1), 5 ng/mL vascular endothelial growth factor (VEGF), 5 ng/mL heparin, 5 ng/mL ascorbic acid, nonessential amino acids, *β*-mercaptoethanol, 1x Penicillin, and 1x streptomycin. All Sca-1+ cells used for this study were between passages 2 and 5.

### 2.2. Western Blotting

Protein samples (30 *μ*g) were separated on SDS-polyacrylamide gels by electrophoresis and the proteins were transferred to immobilon-P membranes (GE Healthcare). Level of the ALDH1 protein was determined using anti-ALDH1A1 antibodies (Abcam) at a concentration of 1 : 1000. Anti-enolase antibody (Santa Cruz Biotechnology, Santa Cruz) was used as housekeeping marker at a concentration of 1 : 1000. The bound antibodies were visualized with horseradish peroxidase- (HRP-) coupled secondary antibody and chemiluminiscence detection system.

### 2.3. ALDH Activity Assay by Spectrophotometer

ALDH1 activity was measured in unsorted control and ALDH specific inhibitor, diethylaminobenzaldehyde- (DEAB-) treated cells by following the procedure as described elsewhere [6]. In brief, enzymatic activity of ALDH1 in cell lysate was determined spectrophotometrically by reductive reaction of NAD+ to NADH at 340 nm (Beckman).

10 mM phenyl acetaldehyde was used as the substrate for ALDH1. All the assays were carried out at 25°C in 0.1 M sodium pyrophosphate buffer, pH = 9.5. 2.4 mM NAD+.

### 2.4. Flow Cytometry Based Sorting of ALDH^high^ and ALDH^low^ Sca-1+ Cells

Sca-1+ cells were sorted by flow cytometry into subpopulations having high or low ALDH enzyme activity using the Aldefluor kit (Stem Cell Technologies, Vancouver, Canada), as per manufacturer's instructions. Briefly, cultured human fetal Sca-1+ cells were resuspended in Aldefluor assay buffer, and 1 × 10^6^ cells/mL cell suspension was added to a tube containing 5 *µ*L of the ALDH substrate, Aldefluor. 0.5 mL of this suspension was transferred to a second tube, containing the DEAB, which serves as a negative control. Following 30-minute incubation at 37°C and centrifugation, the cells were washed with the assay buffer, followed by resuspension in 0.3 mL of ice cold Aldefluor assay buffer for flow sorting (carried out on BD FACS Aria). Viability of the cells was determined by 7-amino actinomycin D (7-AAD). The DEAB negative control was used to set the gate. Cells having the highest fluorescence at 515 nm were collected as the ALDH^high^ population. The nonfluorescent population at 515 nm was collected as the ALDH^low^ cells. After sorting, the ALDH^high^ and ALDH^low^ Sca-1+ cells were cultured under identical culture conditions. After one week in culture, Aldefluor assay was carried out on the two populations as described above, followed by flow cytometric analysis of ALDH activity.

### 2.5. Quantitative (Real-Time) Reverse Transcriptase (RT) PCR (qRT-PCR)

Equal number of ALDH^high^ and ALDH^low^ cells at passage 2 was seeded in a 6-well plate and was harvested at 70–80% confluency. The cell pellet obtained after centrifugation was used for RNA isolation. Total RNA was purified from the ALDH^high^ and ALDH^low^ Sca-1+ cells using RNeasy Mini kit (Qiagen). 1 *μ*g of total RNA was reverse transcribed using high capacity cDNA transcription kit (Applied Biosystems), followed by high throughput quantitative gene expression analysis using Taqman probes for all the genes analyzed. To assess the status of stemness markers, the following Taqman probes were used:* Oct4* (Hs00982625_m1) for organic cation transporter-4 gene,* Nanog* (Hs02387400_g1) for the gene of nanog homeobox,* GATA4* (Hs00171403_m1) for GATA binding protein 4 gene,* Isl1* (Hs00158126_m1) for* ISL1* transcription factor gene, and* MEF2C* (Hs00231149_m1) for myocyte enhancer factor 2C gene. Expression of two genes, the nuclear antigen* Ki67* (Hs01032443_m1) and the antiapoptotic factor, B-cell CLL/lymphoma (*Bcl*) 2 (Hs00608023_m1) were assessed for comparative analysis of proliferation and cell death, respectively. The assay was carried out on the StepOne Plus Real-Time PCR platform (Applied Biosystems). 18S was used as the internal control gene for all samples and the ΔΔCt method was used to calculate fold change of gene expression.

### 2.6. Cell Death Assay after Aldehydic Challenge

First ALDH^high^ and ALDH^low^ Sca-1+ cells were treated with 50 *μ*M acetaldehyde for 4 hr. Acetaldehyde-induced apoptotic cell death was determined using annexin V staining via flow cytometry following manufacturer's instructions (Roche).

### 2.7. Statistical Analysis

All experiments were done in triplicate. FACS and real-time PCR data were analyzed using Graphpad Prism (GraphPad Software, Inc., CA, USA). All data have been presented as mean ± standard error over mean (SEM). For analysis of the FACS data, 1-way ANOVA, followed by Kruskal-Wallis test was used to calculate significance of the differences. For analysis of the RT-PCR data, unpaired *t*-test was used to compare significance of differences between ALDH^high^ and ALDH^low^ cells for each individual gene. In all cases, differences were considered significant at *P* values <0.05.

## 3. Results

### 3.1. ALDH1 Level and Activity in Cultured Sca-1+ Human Fetal Cells

**Figure 1:**
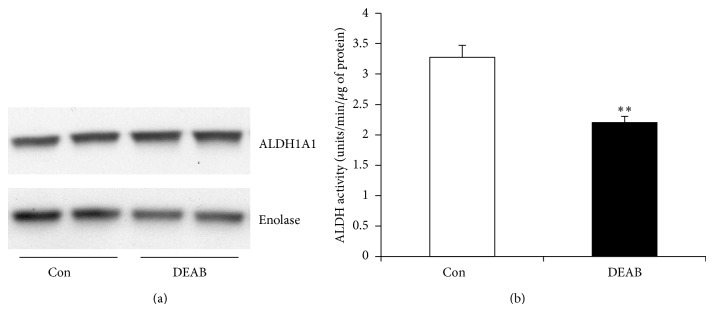
Level and activity of ALDH1. (a) Western immunoblot bands of ALDH1 and enolase, the loading control for cytosolic proteins, and (b) measurement of ALDH activity with phenyl acetaldehyde as substrate from total cell lysates (below) of control (open bar) and DEAB (1.5 *μ*M) treated (filled bar) cells. ^**^ *P* value < 0.006.

Prior to isolating ALDH^high^ cells using Aldefluor kit, which is based on ALDH1 activity, we first determined ALDH1 presence/level in cultured human fetal cells by Western blot analysis. The results showed that these cells do express ALDH1A1 (Figure 1(a)). We also found significant ALDH1 activity in human fetal cell lysates by spectrophotometric assay using phenyl acetaldehyde as substrate (Figure 1(b)). In this activity assay, conversion of phenyl acetaldehyde into phenyl acetic acid by ALDH, generating NADH was measured spectrophotometrically at 340 nm. We found that DEAB (1.5 *μ*M for 1 hour at 37°C) significantly decreased ALDH1 activity.

### 3.2. Identification and Isolation of ALDH^high^ Cells

**Figure 2:**
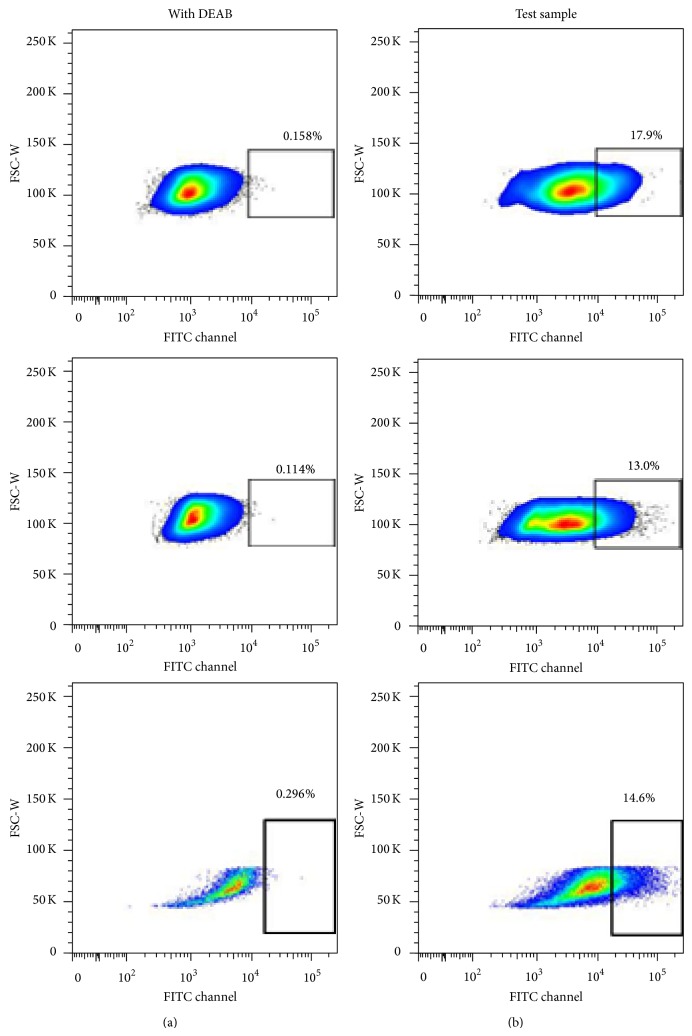
Identification of ALDH^high^ subpopulation in cultured fetal Sca-1+ cells using activity based flow cytometric sorting. There is a distinct and consistent ALDH^high^ Sca-1+ cell population (10–15%) in fetal heart. Dot plots have been shown for three human fetal Sca-1+ cell samples. Left panel represents negative control for each sample. Right panel is the test sample in the absence of the inhibitor, DEAB. ALDH activity is measured in the FITC channel, represented along *x*-axis; forward scatter (FSC) is on the *y*-axis.

We went ahead to identify and isolate the Sca-1+ human fetal cells subpopulation with the highest level of ALDH1 activity using the fluorescence based Aldefluor assay, which has been routinely utilized to identify and isolate primitive hematopoietic stem cells [7]. Using this assay, coupled with flow cytometry, we identified a subpopulation of ~15% cells within the Sca-1+ cells which demonstrated ALDH^high^ phenotype (Figure 2). Close to 98% of cells were viable with this procedure as determined by 7-AAD staining (data not shown). Both the ALDH^high^ and ALDH^low^ cells were sorted and expanded for downstream characterization.

### 3.3. ALDH^high^ Sca-1+ Cells Are Enriched in Stemness and Cardiac Development-Specific Genes

**Figure 3:**
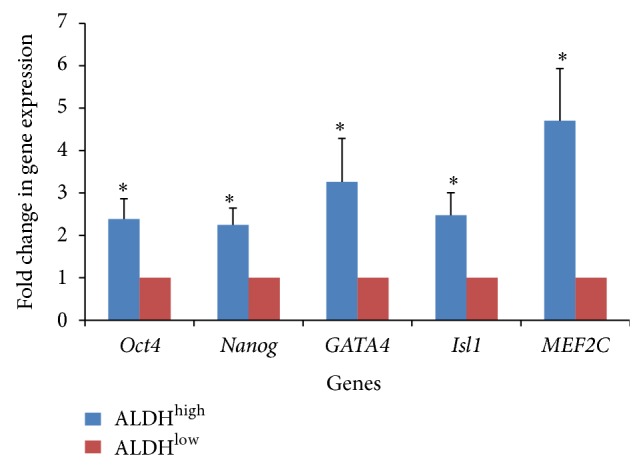
Real-time PCR analysis of ALDH^high^ and ALDH^low^ Sca-1+ cells. Increased expression of markers for stem cells, early cardiac, and self-renewal exclusively in ALDH^high^ cells compared to ALDH^low^ Sca-1+ cells. (*n* = 4; ^*^ *P* value < 0.05).

Equal number of first passage ALDH^high^ and ALDH^low^ Sca-1+ cells were seeded under identical culture conditions and harvested for real-time PCR after 48–72 hrs. ALDH^high^ cells demonstrated relatively high levels of stem cell-specific markers such as* Oct4* and* Nanog* as compared to ALDH^low^ Sca-1+ cells (*P* < 0.05) (Figure 3). In addition, multiple early mesoderm-specific transcripts, such as* GATA4*,* Isl1* and* MEF2C*, were also enriched in ALDH^high^ Sca-1+ cells compared to ALDH^low^ Sca-1+ cells (*P* value < 0.05) (Figure 3).

### 3.4. Increase in Proliferation and Survival Potency in ALDH^high^ Sca-1+ Cells

**Figure 4:**
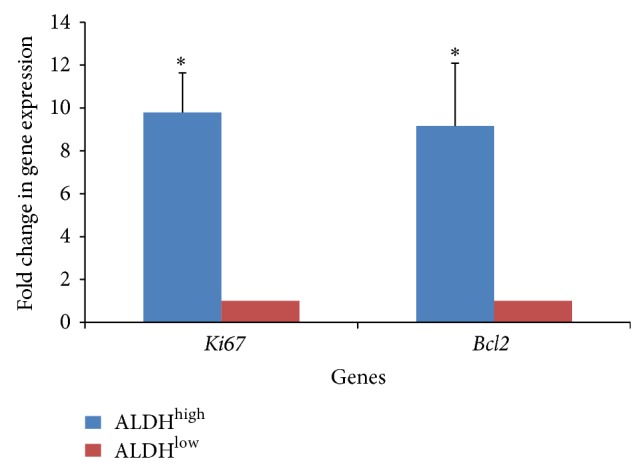
Gene expression analysis of proliferation and prosurvival genes in ALDH^high^ and ALDH^low^ Sca-1+ cells. Quantitative real-time PCR based gene expression analysis of a proliferation-specific gene (*Ki67*) and antiapoptotic gene,* Bcl2* to compare ALDH^high^ and ALDH^low^ Sca-1+ cells. Fold change in gene expression is with respect to ALDH^low^ Sca-1+ cells. (*n* = 3; ^*^ *P* value < 0.05).

We also observed a difference in survival and proliferation status between the two populations, immediately after sorting and seeding them. The ALDH^low^ Sca-1+ cells had a large proportion of floating, dead cells when compared to the ALDH^high^ Sca-1+ cells. In order to confirm this, equal number of ALDH^high^ and ALDH^low^ Sca-1+ cells were seeded and harvested after 48 hours, followed by assessment for expression of a proliferation-specific gene,* Ki67* and an antiapoptotic marker,* Bcl2*. The ALDH^high^ Sca-1+ cells demonstrated significant upregulation of both these markers when compared to the ALDH^low^ Sca-1+ cells (*P* value < 0.05) (Figure 4), indicating enhanced proliferation as well as survival advantage of ALDH^high^ Sca-1+ cells.

The apoptotic cell death was lower in ALDH^high^ Sca-1+ cells compared to ALDH^low^ Sca-1+ cells in both basal condition and acetaldehyde treatment as shown in Table 1.

### 3.5. ALDH^high^ Sca-1+ Cells Produce Increased Number of ALDH^high^ Sca-1+ Cells upon Culture

**Figure 5:**
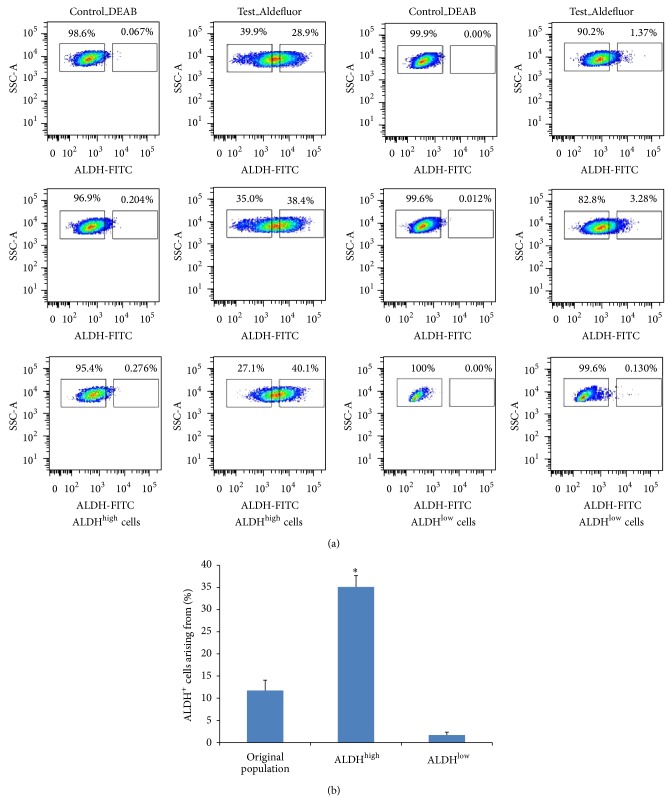
Analysis of the potential of ALDH^high^ and ALDH^low^ Sca-1+ cells to recapitulate the original population. (a) Representation of flow cytometric analysis of ALDH^high^ Sca-1+ cells (left panel) and ALDH^low^ Sca-1+ cells (right panel) one week after* in vitro* culture. (b) Graphical representation of the percentage of ALDH^high^ Sca-1+ cells in the original population, arising from ALDH^high^ Sca-1+ cells and ALDH^low^ Sca-1+ cells. (*n* = 3; ^*^ *P* value < 0.001).

In order to test the potential of ALDH^high^ and ALDH^low^ Sca-1+ cells to give rise to all the cell types existing in the original population from which they were derived, the two populations were cultured. After two weeks in culture, both populations were subject to the Aldefluor assay to determine ALDH activity. While the ALDH^low^ Sca-1+ cells could only give rise to ALDH^low^ subpopulation (>97%; *P* value < 0.05) (Figure 5), the ALDH^high^ Sca-1+ cells recapitulated the original population by giving rise to both the ALDH^high^ and ALDH^low^ populations, in addition to maintaining a high percentage of the cells with ALDH^high^ phenotype. This was consistently observed for multiple samples, and this observation held true even after 2-3 weeks of culture.

## 4. Discussion

This is the first study which identifies a distinct subpopulation of cells in the fetal human heart having high ALDH activity and possessing enhanced potency for self-renewal, proliferation, survival and multipotency.

ALDH has been shown to be a functional marker of stem cells in multiple tissues and tumors of multiple organs [8–11]. The earliest studies of identification and isolation of live ALDH^high^ cells using a fluorescence based assay were carried out in cord blood cells [7] and gradually expanded to other types of stem cells [12–14]. In this* in vivo* cellular assay, BODIPY-aminoacetaldehyde (BAAA) is converted by intracellular isoform of ALDH, ALDH1 into a negatively charged reaction product, BODIPY-aminoacetate (BAA) which is retained inside cells, causing the cells expressing high levels of ALDH to become brightly fluorescent. Now this method, known as the “Aldefluor assay” is used routinely to enrich ALDH^high^ stem/progenitor cells from multiple tissues and tumor samples [9].

We confirmed biochemically that ALDH1 was expressed in human fetal Sca-1+ cells using immunoblotting technique and significant ALDH1 activity was found by enzymatic activity assay when phenyl acetaldehyde was used as a substrate (Figure 1). After confirming that human fetal Sca-1+ cells contain ALDH1, we used flow cytometry based Aldefluor assay to identify and sort the ALDH^high^ subpopulation. This fraction comprised ~15% of the human fetal heart cells (Figure 2). This is higher than the ALDH^high^ percentage reported from other tissues [15–17], that is, 0.5–5%. This increase in ALDH^high^ population may be because of fetal origin. Increase in numbers of cardiac progenitors in neonatal heart compared to adult heart has been reported in earlier studies [18, 19]. Further studies could demonstrate whether this is true for ALDH^high^ Sca-1+ cells too.

In addition to upregulation of typical stem cell markers (Oct4 and Nanog) in the ALDH^high^ Sca-1+ cells with respect to ALDH^low^ Sca-1+ cells, the Isl1 transcript, which is a well-established marker during cardiogenesis in the fetus, was seen to be enriched in the ALDH^high^ Sca-1+ cells. Another interesting observation in our study was the significant enrichment of MEF2C transcription factor exclusively in the ALDH^high^ Sca-1+ cells. MEF2C is known to be one of the earliest markers of cardiac lineage [20], validating the hypothesis that ALDH^high^ Sca-1+ cells might represent a primitive cardiac population in the fetal heart.

We observed higher expression of the proliferative marker Ki67 and antiapoptotic transcript Bcl2 in ALDH^high^ Sca-1+ cells. Considering that primitive progenitor cells typically possess high proliferative potential, this indicates yet again that ALDH^high^ Sca-1+ cells might represent the transit amplifying cells in the fetal heart, which proliferate extensively, and have robust prosurvival mechanisms, as indicated by the Bcl2 levels. The role of Bcl2 in stem cell survival and function has been extensively documented in hematopoietic stem cells (HSCs) [21, 22], intestinal stem cells [23], and melanocyte stem cells [24], to name a few. It has been shown that Bcl2 increases both number and self-renewal potential of HSCs by inhibiting their apoptosis [21]. In a study of embryonic stem cell (ESC) transplantation after cerebral ischemia in rats, overexpression of Bcl2 in the ESCs increased survival of the transplanted cells and brought about significant functional recovery in the brain [25]. In fact, acetaldehyde-induced apoptosis was less in ALDH^high^ Sca-1+ cells with respect to ALDH^low^ Sca-1+ cells.

Upon subsequent expansion, ALDH^high^ Sca-1+ cells gave rise to all population of cells including 28 to 40% of ALDH^high^ Sca-1+ cells and remaining ALDH^low^ Sca-1+ cells, thereby recapitulating the original population; however ALDH^low^ Sca-1+ cells primarily gave rise to ALDH^low^ Sca-1+ cells in culture (<3.5% of ALDH^high^ Sca-1+ cells). It was shown in case of prostate stem cells that ALDH^high^ cells proliferate much more efficiently compared to ALDH^low^ cells [8].

In accordance with earlier studies, our observations demonstrate that ALDH^high^ Sca-1+ cells represent a primitive stem/progenitor cell population in the human fetal heart. Even though existence of ALDH^high^ Sca-1+ cells in adult human heart remains to be addressed, based on our findings, we hypothesize that the adult heart also harbors an ALDH^high^ subpopulation, though their number might be much lower than the fetal ones. It would therefore be interesting to follow up this study in adult human heart samples and also investigate if there is a change in the number or characteristics of ALDH^high^ Sca-1+ cells in pathological heart samples.

In recent years, bone marrow-derived ALDH^high^ cells were recruited to the area of ischemia within hours of transplantation and continued accumulating for up to 1 week, increasing capillary formation in a hind limb ischemia model [16]. ALDH^high^ cell treatment enhanced the perfusion ratio continuously even after 3 weeks compared to ALDH^low^ cells [16]. In another study, human umbilical cord blood (UCB) derived ALDH^high^ cells with nanoparticle label were injected into mice 24 hrs after induction of myocardial infarction. The ALDH^high^ cells, but not the ALDH^low^ cells, preferentially homed to and increased blood vessel formation in the infarcted host myocardium [26]. These studies have provided a strong basis for the clinical potential of ALDH^high^ cells in regenerative medicine. In fact, two clinical trials were launched based on these studies [27, 28]. Therefore, our current study of identifying and partially characterizing ALDH^high^ Sca-1+ progenitor cells in human fetal heart is an important first step in moving forward to address multiple questions, including differentiation studies of these cells, understanding the role of different ALDH isozymes in the function of Sca-1+ cells, and testing the therapeutic potential of ALDH^high^ progenitor cells in preclinical models of cardiovascular diseases. More importantly, extrapolating this approach to other types of cardiac progenitor cells such as c-kit+ cells would be very useful for the advancement of cardiac regenerative medicine. In conclusion, our study reaffirms that high ALDH activity can be used as a functional biochemical marker to isolate progenitor cells within the heart, which possess enhanced self-renewal, proliferation, and survival potential.

## Figures and Tables

**Table 1:** Reduced apoptotic death in ALDH^high^ cells with acetaldehyde treatment.

Cell type	Basal		Acetaldehyde-treated	
	ALDH^high^ cells	ALDH^low^ cells	ALDH^high^ cells	ALDH^low^ cells
% apoptotic death	102 ± 12	197 ± 2^*^	163 ± 32	338 ± 5^∗∗##^

^*^ *P* value < 0.001 versus ALDH^high^ cells in basal conditions; ^**^ *P* value < 0.005 versus ALDH^high^ cells with acetaldehyde treatment; ^##^ *P* value < 0.0001 versus ALDH^low^ cells in basal conditions. *N*: 4 repeats.

## References

[1] Wang H., Chen H., Feng B. (2014). Isolation and characterization of a Sca-1^+^/CD31^−^ progenitor cell lineage derived from mouse heart tissue. *BMC Biotechnology*.

[2] Oh H., Bradfute S. B., Gallardo T. D. (2003). Cardiac progenitor cells from adult myocardium: homing, differentiation, and fusion after infarction. *Proceedings of the National Academy of Sciences of the United States of America*.

[3] Nagai T., Matsuura K., Komuro I. (2013). Cardiac side population cells and sca-1-positive cells. *Methods in Molecular Biology*.

[4] Smits A. M., van Vliet P., Metz C. H. (2009). Human cardiomyocyte progenitor cells differentiate into functional mature cardiomyocytes: an in vitro model for studying human cardiac physiology and pathophysiology. *Nature Protocols*.

[5] Chavakis E., Koyanagi M., Dimmeler S. (2010). Enhancing the outcome of cell therapy for cardiac repair: progress from bench to bedside and back. *Circulation*.

[6] Chen C.-H., Budas G. R., Churchill E. N., Disatnik M.-H., Hurley T. D., Mochly-Rosen D. (2008). Activation of aldehyde dehydrogenase-2 reduces ischemic damage to the heart. *Science*.

[7] Storms R. W., Trujillo A. P., Springer J. B. (1999). Isolation of primitive human hematopoietic progenitors on the basis of aldehyde dehydrogenase activity. *Proceedings of the National Academy of Sciences of the United States of America*.

[8] Burger P. E., Gupta R., Xiong X. (2009). High aldehyde dehydrogenase activity: a novel functional marker of murine prostate stem/progenitor cells. *Stem Cells*.

[9] Balber A. E. (2011). Concise review: aldehyde dehydrogenase bright stem and progenitor cell populations from normal tissues: characteristics, activities, and emerging uses in regenerative medicine. *Stem Cells*.

[10] Ikeda J. I., Mamat S., Tian T. (2012). Reactive oxygen species and aldehyde dehydrogenase activity in Hodgkin lymphoma cells. *Laboratory Investigation*.

[11] Lindahl R. (1992). Aldehyde dehydrogenases and their role in carcinogenesis. *Critical Reviews in Biochemistry and Molecular Biology*.

[12] Armstrong L., Stojkovic M., Dimmick I. (2004). Phenotypic characterization of murine primitive hematopoietic progenitor cells isolated on basis of aldehyde dehydrogenase activity. *Stem Cells*.

[13] Gentry T., Deibert E., Foster S. J., Haley R., Kurtzberg J., Balber A. E. (2007). Isolation of early hematopoietic cells, including megakaryocyte progenitors, in the ALDH-bright cell population of cryopreserved, banked UC blood. *Cytotherapy*.

[14] Gentry T., Foster S., Winstead L., Deibert E., Fiordalisi M., Balber A. (2007). Simultaneous isolation of human BM hematopoietic, endothelial and mesenchymal progenitor cells by flow sorting based on aldehyde dehydrogenase activity: implications for cell therapy. *Cytotherapy*.

[15] Capoccia B. J., Robson D. L., Levac K. D. (2009). Revascularization of ischemic limbs after transplantation of human bone marrow cells with high aldehyde dehydrogenase activity. *Blood*.

[16] Putman D. M., Liu K. Y., Broughton H. C., Bell G. I., Hess D. A. (2012). Umbilical cord blood-derived aldehyde dehydrogenase-expressing progenitor cells promote recovery from acute ischemic injury. *Stem Cells*.

[17] Keller L. H. (2009). Bone marrow-derived aldehyde dehydrogenase-bright stem and progenitor cells for ischemic repair. *Congestive Heart Failure*.

[18] Mishra R., Vijayan K., Colletti E. J. (2011). Characterization and functionality of cardiac progenitor cells in congenital heart patients. *Circulation*.

[19] Simpson D. L., Mishra R., Sharma S., Goh S. K., Deshmukh S., Kaushal S. (2012). A strong regenerative ability of cardiac stem cells derived from neonatal hearts. *Circulation*.

[20] Edmondson D. G., Lyons G. E., Martin J. F., Olson E. N. (1994). Mef2 gene expression marks the cardiac and skeletal muscle lineages during mouse embryogenesis. *Development*.

[21] Domen J., Cheshier S. H., Weissman I. L. (2000). The role of apoptosis in the regulation of hematopoietic stem cells: overexpression of BCL-2 increases both their number and repopulation potential. *The Journal of Experimental Medicine*.

[22] Domen J., Weissman I. L. (2000). Hematopoietic stem cells need two signals to prevent apoptosis; BCL-2 can provide one of these, Kitl/c-Kit signaling the other. *The Journal of Experimental Medicine*.

[23] Potten C. S. (1998). Stem cells in gastrointestinal epithelium: numbers, characteristics and death. *Philosophical Transactions of the Royal Society B: Biological Sciences*.

[24] Nishimura E. K., Granter S. R., Fisher D. E. (2005). Mechanisms of hair graying: incomplete melanocyte stem cell maintenance in the niche. *Science*.

[25] Wei L., Cui L., Snider B. J. (2005). Transplantation of embryonic stem cells overexpressing Bcl-2 promotes functional recovery after transient cerebral ischemia. *Neurobiology of Disease*.

[26] Sondergaard C. S., Hess D. A., Maxwell D. J. (2010). Human cord blood progenitors with high aldehyde dehydrogenase activity improve vascular density in a model of acute myocardial infarction. *Journal of Translational Medicine*.

[27] Perin E. C., Silva G. V., Zheng Y. (2012). Randomized, double-blind pilot study of transendocardial injection of autologous aldehyde dehydrogenase-bright stem cells in patients with ischemic heart failure. *The American Heart Journal*.

[28] Perin E. C., Silva G., Gahremanpour A. (2011). A randomized, controlled study of autologous therapy with bone marrow-derived aldehyde dehydrogenase bright cells in patients with critical limb ischemia. *Catheterization and Cardiovascular Interventions*.

